# Adaptive Choice Biases in Mice and Humans

**DOI:** 10.3389/fnbeh.2020.00099

**Published:** 2020-07-14

**Authors:** Mario Treviño, Ricardo Medina-Coss y León, Belén Haro

**Affiliations:** Laboratorio de Plasticidad Cortical y Aprendizaje Perceptual, Instituto de Neurociencias, Universidad de Guadalajara, Guadalajara, Mexico

**Keywords:** mouse, human, choice-bias, discriminability, two-alternative choice

## Abstract

The contribution of non-sensory information processing to perceptual decision making is not fully understood. Choice biases have been described for mice and humans and are highly prevalent even if they decrease rewarding outcomes. Choice biases are usually reduced by discriminability because stimulus strength directly enables the adjustments in the decision strategies used by decision-makers. However, choice biases could also derive from functional asymmetries in sensory processing, decision making, or both. Here, we tested how particular experimental contingencies influenced the production of choice biases in mice and humans. Our main goal was to establish the tasks and methods to jointly characterize psychometric performance and innate side-choice behavior in mice and humans. We implemented forced and un-forced visual tasks and found that both species displayed stable levels of side-choice biases, forming continuous distributions from low to high levels of choice stereotypy. Interestingly, stimulus discriminability reduced the side-choice biases in forced-choice, but not in free-choice tasks. Choice biases were stable in appearance and intensity across experimental days and could be employed to identify mice and human participants. Additionally, side- and alternating choices could be reinforced for both mice and humans, implying that choice biases were adaptable to non-visual manipulations. Our results highlight the fact that internal and external elements can influence the production of choice biases. Adaptations of our tasks could become a helpful diagnostic tool to detect aberrant levels of choice variability.

## Introduction

Through psychophysics, experimenters can estimate perceptual thresholds of detection and how changes in stimulus strength lead to perceptual changes. However, the exact contribution of non-sensory information processing to perceptual decision making is not fully understood (Ahissar et al., 2009; Resnik et al., 2011; Trevino et al., 2013). For instance, observers can present sensory and non-sensory biases in their choices (Linares et al., 2019). Throughout this work, we will refer to the term *choice bias* simply as the (rational or irrational) tendency to choose one alternative over another. Stereotypical choice behavior exhibits low variability from trial to trial and has no apparent goal or function (Langen et al., 2011; Novak et al., 2016). At the individual level, choice biases can be easily identified as a horizontal shift in the psychometric function relative to the indecision point (Busse et al., 2011). For these psychometric functions, the probability of choosing one alternative is plotted against stimulus discriminability, with the indecision point representing an equal probability of choosing the opposite alternative (i.e., no particular preference). Most importantly, choice biases can produce false perceptual sensitivity thresholds if they are not detected and considered properly (Gold and Ding, 2013).

Choice biases have been described for mice and humans (Busse et al., 2011; Trevino, 2014; Abrahamyan et al., 2016) and are highly prevalent in two-alternative-forced-choice (2AFC) perceptual tasks with symmetric designs, even if they decrease rewarding outcomes (Gold and Ding, 2013). Notably, lowering stimulus discriminability can increase the prevalence of choice biases (Trevino, 2014; Linares et al., 2019). This observation could derive from the simple fact that stimulus strength directly enables the adjustments in the strategies used by decision-makers in 2AFC tasks. At the extreme, with low or zero discriminability, responding exclusively to one side or the other, or even alternating between sides, are equally valid strategies (Killeen et al., 2018). However, an additional consideration is that choice biases could also be influenced by other factors that are not directly linked to the explicit properties of the sensory stimuli. For instance, some decisional biases could derive from functional asymmetries in sensory processing, decision making, or both (Schwartz et al., 2007; Fritsche et al., 2017; Linares et al., 2019). Therefore, establishing a conceptual difference between internal and external factors that influence choice biases becomes a crucial step to understand how normal and abnormal behaviors are organized.

Here, we explored how particular experimental contingencies influenced the production of choice biases in mice and humans. A primary goal was to design tasks across species that could be used translationally to study innate choice biases. For that, we implemented standard two-alternative forced (2AFC) and un-forced (2AUC) visual tasks for mice and humans, with equally rewarded alternatives. In the 2AFC tasks, the discriminative stimulus predicted the side of reinforcement, whereas in the 2AUC tasks, both sides were equally reinforced. Using these adaptations, we found that stimulus similarity increased the side-choice biases in the 2AFC tasks but not in the 2AUC tasks. Furthermore, both groups of mice and humans displayed stable levels of choice biases, forming continuous distributions from low to high levels of choice stereotypy. Although choice biases were stable in appearance and intensity across experimental days, they were also influenced by recent choice and reward histories. Favoring the notion that decisional biases can also be internally mediated, we found that side-biased and alternating choice sequences could be employed to identify mice and human participants with a high degree of certainty. Also, by reinforcing side-preference and alternation, we found that choice biases were strongly adaptable to non-visual manipulations. Altogether, this study established and validated the conditions to jointly characterize psychometric performance and side-choice behavior in mice and humans. Choice biases could become useful biomarkers to diagnose psychopathologies and mental disorders that are characterized by aberrant levels of behavioral variability.

## Materials and Methods

### Animals

We used 2–4-month-old C57BL/6J male mice (18–32 g) housed in groups of up to three animals per polycarbonate cage (Alternative Design, United States; 29.2 × 18.4 × 12.7 cm) under standard laboratory conditions, with unrestricted access to food (Rodent Lab Chow 5001, Purina) and water. The housing room operated with regular light/dark cycle, with constant temperature (22°C ± 2°C) and humidity (55 ± 20%). The cages were changed once per week with fresh sawdust. All experiments were done during the light phase of the day, between 8 a.m. and 6 p.m., from Monday to Friday. Our animal experiments followed the Mexican animal welfare guidelines (SAGARPA, NOM-062-ZOO-1999) and were approved by the ethics committee of our institution (ET062018-265 and ET112019-290; Instituto de Neurociencias, Universidad de Guadalajara, Mexico).

### Visual Task for Mice

**FIGURE 1:**
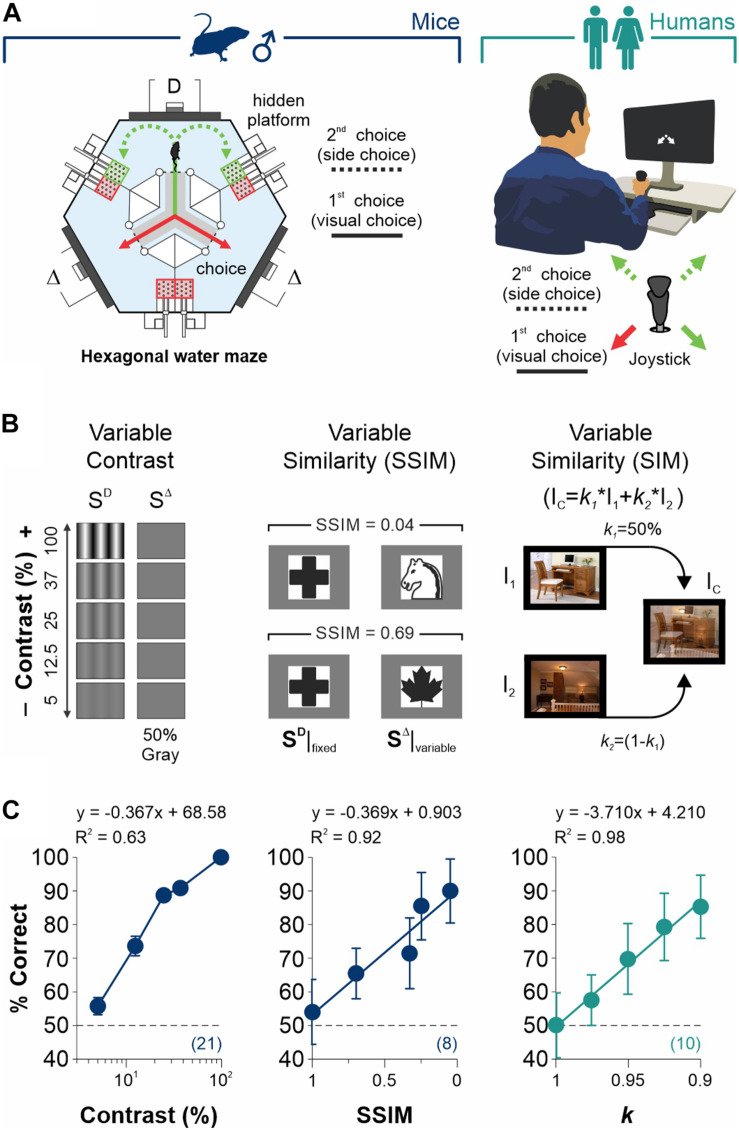
Measurement of discriminative capacities in mice and humans. **(A)** Visual tasks employed to measure discriminative choices and decisional biases in male mice (left) and humans (right). Each trial is composed by (i) a discriminative forced-choice (visual choice, solid lines) and (ii) a side-choice (free choice, dashed lines). **(B)** Scalable difficulty of discrimination by increasing stimulus contrast (left panel), stimulus similarity (SSIM, center), or image similarity (right). **(C)**% Correct choices (% Correct) for mice (navy blue) and humans (dark cyan) as a function of stimulus contrast (left panel), SSIM (center), or image similarity (right). Regressions and coefficients of determination on top of each panel. All experiments throughout this work involved well-trained mice that exhibited ≥ 90% correct choice performance in the grating discrimination task (100% contrast). Number of subjects in parentheses.

For our visual experiments with mice, we used an automated forced/unforced-choice (2AFC/2AUC, see below) aquatic discrimination task that we described in detail recently (Trevino et al., 2018). The apparatus consisted of a hexagonal glass pool with a decision chamber at the center, giving access to three interior arms facing computer-controlled monitors (left panel, Figure 1A). Adjacent to each side of the three dividers separating each arm, we placed one of six computer-controlled acrylic platforms (8 cm long, 8 cm wide, 18 cm high). Each platform was controlled independently to lower or raise just below the water surface. We filled the pool with tap water (21°C ± 1°C) to reach a level 1 cm above the elevated platforms. To train the mice, we released them into the pool, starting from one random elevated platform. During consecutive trials, they learned to swim toward the discriminative conditioned stimulus S^D^ because it predicted the existence of two elevated platforms placed to the left and right sides of that arm. Animals choosing the ‘correct arm’ displaying the S^D^ were allowed to rest on the elevated platform for 40 s, but they could rest only for 10 s after choosing the ‘wrong arm’. The arm projecting the S^D^ was selected pseudo-randomly on each trial, but it could not repeat over consecutive trials (Herrera and Trevino, 2015).

Visual performance was measured as the group average correct choices/mouse, whereas the escape latencies (in s) were taken as the time it took the animal to reach an escape platform. The overall training procedures were identical to those described previously and concluded when the mice achieved ≥ 90% correct choices for two consecutive days (Trevino et al., 2018). We characterized visual contrast responses by using static sine-wave gratings (0.04 cycles/degree) with multiple contrasts. Our screens were gamma-corrected to ensure linearly increasing intensity scales (Trevino et al., 2019). We also tested the discriminative capacities of the mice by using a set of equiluminant images with variable structural similarity among them (SSIM). The SSIM measures image quality by using structural similarity between target and reference images (Trevino et al., 2013). We conducted the experiments inside a quiet room under photopic conditions (230 lux ± 2.5 lux at 24 cm from the monitors). For all experiments performed and reported throughout this work, we used well-trained mice that exhibited ≥90% correct choice performance in the grating discrimination task with 100% contrast (Trevino et al., 2018).

### Human Participants

We performed experiments with 55 healthy volunteers (26 men and 29 women). Their mean age was 23.9 ± 0.7 years (a minimum of 16, a maximum of 39, mode of 19). Most participants were right-handed (≥90%), with normal or corrected vision, and without detectable neurological disorders or history of drug abuse. We obtained written consent from all participants. The ethics committee of our institution approved all these procedures (ET092018-271; Instituto de Neurociencias, Universidad de Guadalajara, Mexico).

### Visual Task for Humans

Participants sat upright on a stool (with adjustable height) at a desk in front of a manipulandum centered at the midline of a 19-inch computer monitor (right panel, Figure 1A). We recorded the visual responses of the participants using a 17 cm commercial joystick (ThrustMaster 2960623 USB Joystick; sampling rate: 1000 Hz). We instructed the participants to hold the joystick with their dominant hand and grasp the handle with all their fingers. To make a selection, they had to push the handle in the direction specified by arrows projected on the monitor screen, while maintaining their fixation on the center of the screen. We placed the choice regions on the corners of the rectangular search space from the projecting screen. Each choice option occupied 5% of the overall search space, making the response detection sufficiently precise to prevent errors caused by involuntary movement of the joystick. After responding, the participants had to let the joystick return to its initial position (at the center of the screen). The movements of the joystick mapped linearly onto the search space and were recorded and digitized using a standard computer (Intel^®^ Xeon^®^ @ 3.40 GHz; 64 Bit Operating system; Graphics card: NVIDIA Quadro K600, 8 GB). We measured the response times (RT, in s) as the interval between the appearance of the visual stimuli and the moment when the participants placed the joystick in the appropriate response regions.

To investigate discriminative choices and choice biases in humans, we implemented a 2AFC match-to-sample task. During a first forced-choice phase, the subjects had to make a discriminative choice based on identifying the location of a sample stimulus (S^D^) projected during 1 s to the left or right side of a distractor (the S^D^). Concluding the projection of both stimuli, two white arrows appeared on the center of the screen, pointing toward the right and left inferior corners, respectively, indicating the two response options. These arrows remained on the screen until the participant responded. Next, the S^D^ was projected at the center of the screen for either 750 ms or 3 s if the participant responded correctly or incorrectly, respectively (i.e., a 1:4 relationship in the waiting intervals). During the second choice phase, the participants could select left or right sides with forced (2AFC, same 1:4 relationship in waiting intervals) or free (2AUC) side choices, respectively. Here, participants could visualize two white arrows that appeared on the center of the screen, pointing toward the right and left superior corners, indicating the two response options (right panel, Figure 1A). The visual stimuli (415.68 × 301.44 pixels; visual angle: 7° × 5°) consisted of two images (randomly positioned to the left and right sides of the screen). These stimuli had low semantic attributes (image #369 for the S^D^ and #471 for the S^D^; Xu et al., 2014) and were projected on a 27-inch computer screen (Dell P2414H, 1920 × 1080 pixels @ 60 Hz; field of view: 36.3° × 26.2° at a viewing distance of 60 cm). We controlled the difficulty of the discrimination task by creating distractor images with different degrees of similarity (SIM) relative to the discriminative stimulus. We did this by using linear combinations of the two source images (using Matlab function ‘imlincomb’). We gave all participants written instructions on the task, and they performed the experiments in silence. Each session consisted of four blocks of 250 trials, with a 5 min break between blocks. We used programs written in MATLAB R2016a (MathWorks, Inc.; Natick, United States) using the Psychophysics Toolbox extensions (PTB-3) to project the visual stimuli.

### Common Task Designs for Mice and Humans

We implemented 2AFC tasks for mice and humans, where the discriminative stimulus predicted the side of reinforcement: (1) in the ‘correct arm’ for the mouse task or (2) within the lower portion of the stimulus-projection search space for the human task. We extended this task design to include a second 2AUC phase in which we measured unforced-choices (i.e., [2AFC→ 2AUC]). In the 2AUC phase, both sides were equally reinforced and, therefore, the discriminative stimulus did not predict the side of reinforcement: (1) already inside the ‘correct arm’ for the mouse task or (2) within the upper portion of the projecting screen for the human task. Thus, in the [2AFC→ 2AUC] tasks, the discriminative stimulus predicted reinforcement for the first visual choice (‘correct arm’ for mice and ‘correct lower side’ for humans), but provided no information about the second side-choice (left or right side-choice). In experiments illustrated in Figure 7, we adapted the 2AFC task to reinforce alternating or side-choice sequences during the second phase of the task (all experiments carried out in well-trained mice and humans). We employed stimuli with high discriminability (for mice: a grating stimulus with 100% contrast and 0.04 cycles/degree; for humans: original images with *k* = 0). For mouse experiments, we kept the training conditions fixed across pairs of experimental days (i.e., each training block consisted of ≥130 trials/condition), whereas for human experiments, we switched contingencies every 60 trials with a total of 12 blocks/day.

### Analysis of Choice Behavior

Both mice and human participants could select left or right sides during the second forced/free-choice phase of our experiments. They typically displayed a choice bias toward one preferred side (Wilke et al., 2012). To graphically represent such decisional biases, we plotted the trials with the left or right responses as white or black rectangles, respectively. To quantify side preferences, we measured the probability that sequences of choices were produced toward (i) the same side (i.e., complementary sequences of left or right choices) or (ii) in alternation (‘LRL…’ + ‘RLR…’), as previously described (Trevino et al., 2013; Trevino, 2014). In the experiments illustrated in Figure 7, we reinforced single-side or alternating responses by using a training paradigm in which the contingencies favored systematic side-choices (left or right), or systematic alternation.

### Logistic Regression Analysis of Behavior

To estimate the contributions of previous visual choices, side-choices, and their outcomes (success or failure) on the production of current side-choices, we carried out multiple logistic regression model (MLRM) analyses, as described before (Trevino, 2014; Herrera and Trevino, 2015). For mice’s analyses, we also included the position of the S^D^ and the chosen platform as potential predictors of current choice behavior. The scalar coefficients were fit using MATLAB function ‘glmfit.m.’

### Statistical Analysis

We used *t*-tests and one-way ANOVA tests for performance comparisons and repeated measures ANOVA (RM-ANOVA) tests with Bonferroni’s or Wilcoxon Signed Rank *post hoc* tests for group comparisons. We compared the probability distributions using Kolmogorov–Smirnov (KS) tests. We illustrate our group data as averages ± SEM with a significance set at *P* ≤ 0.05.

## Results

### Measurement of Discriminative and Free-Choice Behavior

We first established the conditions to sequentially measure discrimination performance and choice biases in well-trained mice and humans (Figure 1A). More specifically, we designed our visual tasks to measure forced and un-forced/free choice components during two consecutive phases on each trial. The first phase involved a simple discriminative two-alternative forced-choice (2AFC) task. A second phase involved either a forced (2AFC) or an unforced (free choice, 2AUC) task, depending on whether the discriminative stimulus (S^D^) predicted the reinforcer side. We controlled the difficulty of the perceptual task through scalable contrast, structural similarity (SSIM), and similarity (SIM) between the target S^D^ and distractor S^D^ stimuli (see section “Materials and Methods”; Figure 1B). Next, we tested the discriminability of the stimuli through behavioral experiments. As expected, we found that increasing contrast (slope different from zero, *F* = 64.2, *P* < 0.01, *n* = 21; navy blue dots, left panel, Figure 1C) and decreasing stimulus similarity led to a robust increase in visual performance of the mice (SSIM, *F* = 35.3, *P* < 0.01, *n* = 8; center panel, Figure 1C) and humans (*k*, *F* = 273, *P* < 0.001, *n* = 10; dark cyan dots, right panel, Figure 1C). These results illustrate how we controlled the perceptual performance of mice and humans by varying stimulus contrast or similarity (see also Supplementary Figure S1).

### Stimulus Discriminability Reduces the Number of Biased Choices in Forced-Choice Tasks

**FIGURE 2:**
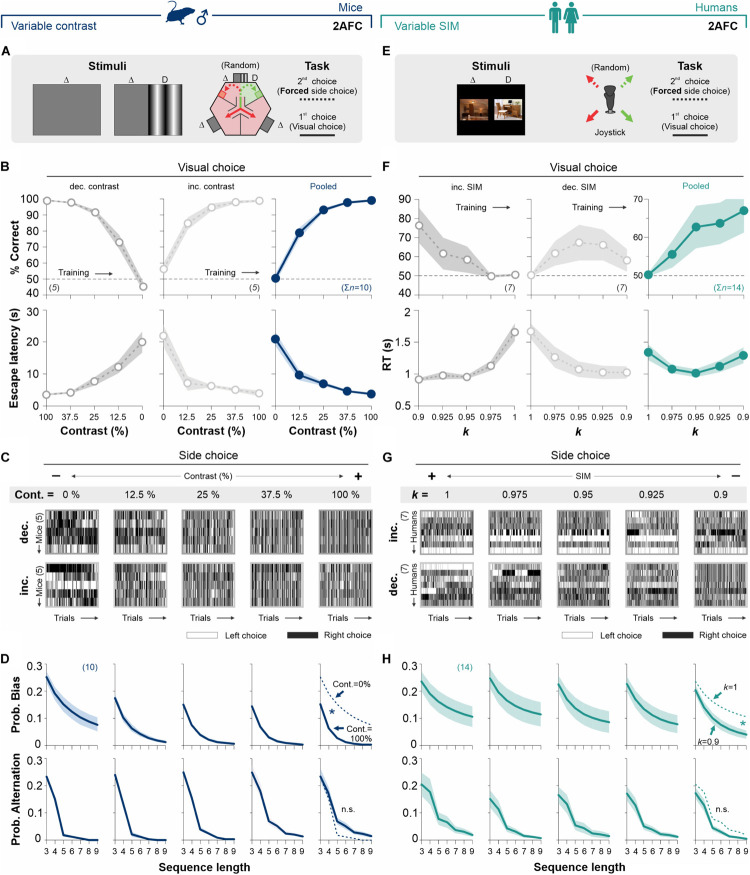
Stimulus discriminability reduces decisional biases in forced-choice tasks. **(A)** The basic version of the mouse task is composed of a two-alternative force choice (2AFC) task where the S^D^ side predicts the location of an escape platform. **(B)**% Correct choices and escape latencies as a function of stimulus contrast for two balanced groups of male mice trained with either decreasing (left panels, 50% gray) or increasing (center panels, 25% gray) contrasts. Panels on the right show averaged data from both groups (navy blue). **(C)** The side-choices of individual mice are illustrated as colormaps with black (right) or white (left) rectangles (i.e., trials) across experimental days. **(D)** Probability of biased (upper row) and alternating (lower row) sequences as a function of sequence length. **(E)** A similar 2AFC task for humans, where the S^D^ side predicts reinforcement. **(F)%** Correct choices and response times for participants trained with decreasing (left panels, in 50% gray) or increasing (center panels, 25% gray) similarity. Pooled data in dark cyan (right panels). **(G)** Side-choice colormaps for humans. **(H)** Biased and alternating side-choice probabilities for humans. Note how increasing stimulus discriminability in both 2AFC tasks decreased the decisional biases in mice (navy blue, **D**) and humans (dark cyan, **H**). Number of subjects in parentheses.

Mice can display choice biases in 2AFC tasks where the sides of reinforced alternatives are randomized and balanced, even if these choices worsen their performance. For simplicity, we define such choice biases as a (rational or irrational) tendency to choose one alternative over another. Notably, although side-choice preferences in these tasks are heterogeneous across animals, they are strongly sensitive to changes in stimulus discriminability (Trevino, 2014). Thus, we first aimed to explore if we could replicate the dependence of decisional biases to stimulus discriminability in our new tasks. We implemented basic versions of a 2AFC task for mice (Figure 2A) and humans (Figure 2E), where the side of the S^D^ predicted access to a positive reinforcer for both visual and side-choices. We counterbalanced our experiments and tested two subgroups of mice (five mice each) by using gratings with decreasing (50% gray dots, left panel) and increasing (25% gray dots, center panel, Figure 2B) contrast levels each day, respectively. As expected, discrimination performance increased with stimulus contrast leading to similar discrimination performances (RM ANOVA test, correct choices: *F* = 2.23, *P* = 0.09; *n* = 10) and escape latencies (escape latencies: *F* = 1.18, *P* = 0.34) for both subgroups of mice (Supplementary Table S1). We pooled together these results to illustrate how the average performance increased with higher stimulus strength (navy blue dots in panels on the right, Figure 2B).

Next, we explored how stimulus contrast influenced choice biases in the 2AFC task (Trevino, 2014). To visualize the side-choice preferences of each mouse, we employed a colormap representation in which we plotted left and right choices as white or black rectangles, respectively, with the testing trials along the *x*-axis (Figure 2C). We then measured the probability of side-biased and alternating choice sequences of different lengths in the choice records of the mice (see section “Materials and Methods”). We found that increasing stimulus contrast from 0 to 100% decreased the decisional biases by ∼74% (area under the curve, AUC; w. differences in the distributions from non-contiguous contrasts categories, Kolmogorov–Smirnov test, *P* < 0.05 for all cases; RM ANOVA test, *F* = 3.94, *P* ≤ 0.004; upper panels, Figure 2D). Alternation probabilities were relatively similar across all contrasts (KS test, *P* ≥ 0.13 for all groups; RM ANOVA test, *F* = 2.18, *P* < 0.001; lower panels, Figure 2D).

Retaking a counterbalanced experimental design, we next characterized the changes in visual performance for human participants (seven participants per group), tested in a 2AFC task with increasing (50% gray dots, Figure 2F) or decreasing (25% gray dots, Figure 2F) stimulus similarity (left and center panels, Figure 2F; see also Supplementary Table S1). Both subgroups of humans had similar visual discrimination performances (RM ANOVA test, correct choices: *F* = 1.82, *P* = 0.14; RT: *F* = 3.77, *P* = 0.06; *n* = 10). The pooled data illustrates how stimulus discriminability influenced human visual performance (dark cyan dots, right panel, Figure 2F). Similar to our previous findings with mice, decreasing stimulus similarity (SIM) reduced the decisional biases by 60% (area under the curve, AUC; differences in [*k* = 1] vs. [*k* = 0.9]; KS test, *P* = 0.027; upper panels Figures 2G,H). Alternation probabilities for humans were also similar across all discriminative conditions (KS test, *P* ≥ 0.42 for all groups; RM ANOVA test, *F* = 0.53, *P* = 0.92; lower panels, Figure 2H). Altogether, these results show how stimulus discriminability reduced choice biases in 2AFC tasks for mice and humans (see also Supplementary Figure S2).

### Discriminability Does Not Influence Choice Biases in Free-Choice Tasks

**FIGURE 3:**
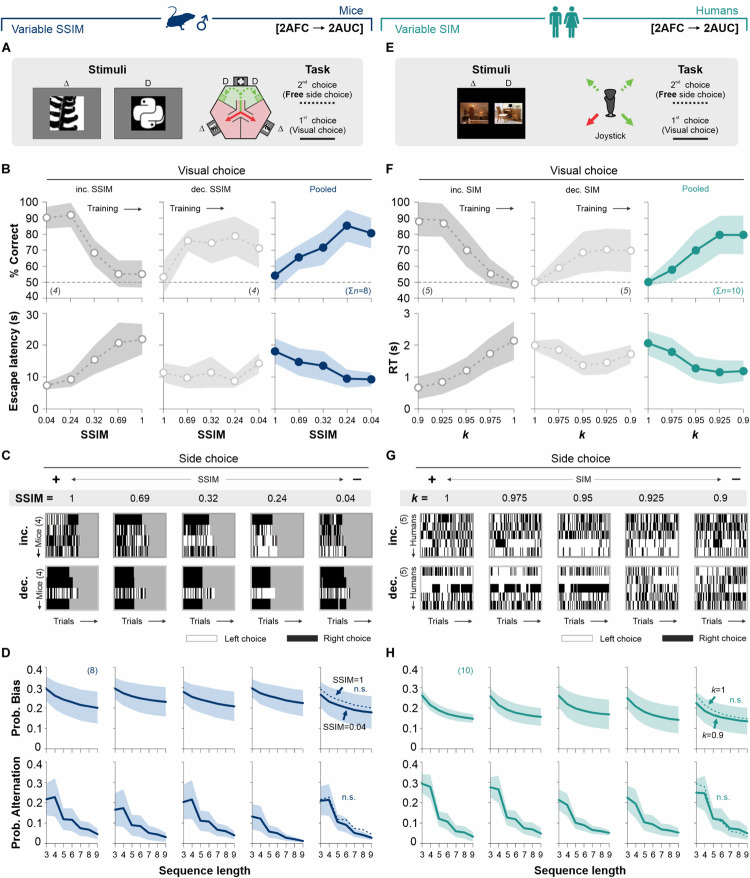
Biased choices are insensitive to changes in stimulus discriminability in free-choice tasks. **(A)** Adaptation of the original mouse task into a two-alternative unforced-choice (2AUC) task where the mice can choose their preferred side (left or right) without any differential implication. Psychometric curves **(B)**, side-choice colormaps **(C),** and biased and alternating side-choice probabilities **(D)** are arranged as in Figure 2. **(E)** Adaptation of the 2AUC task for humans where they can freely choose their preferred side irrespective of where the S^D^ was previously shown. Panels **(F,G,H)** arranged as in Figure 2. Note how stimulus discriminability in the 2AUC tasks did not influence the production of decisional biases in mice (navy blue, **D**) and humans (dark cyan, **H**). Same color coding as in Figure 2. Number of subjects in parentheses.

It is well known that stimuli that drive actions in some contexts can be ineffective in doing so when reinforcement or their predictive value are removed. We reasoned that stimulus discriminability should lose control over the production of choice biases after removing the predictive value of the S^D^. To test this idea, we modified our previous 2AFC tasks to include a second phase in which we measured free choices (i.e., we coupled a 2AUC task during the second side-choice phase) in such a way that access to either left or right reinforcer was always available and, therefore, identical (we will refer to it as the ‘[2AFC→ 2AUC] task’). We tested two subgroups of mice (counterbalanced design: four mice each subgroup) with increasing (50% gray dots, panels on the left, Figures 3A,B) and decreasing similarity (SSIM, 25% gray dots, center panels, Figure 3B; see also Supplementary Table S2). Training with increasing and decreasing SSIMs led to some performance differences between groups (RM ANOVA test, correct choices: *F* = 12.70, *P* < 0.001; escape latencies: *F* = 7.02, *P* < 0.002; *n* = 8), in agreement with previous findings (Trevino et al., 2013). In the right panels of Figure 3B, we illustrate how the average discriminative behavior for all mice improved with decreasing SSIM values. Interestingly, despite the fact that SSIM strongly controlled the visual discrimination performance in this task, we found that choice bias (Kolmogorov–Smirnov test, *P* > 0.12 for all cases; RM ANOVA test, *F* = 0.05, *P* > 0.5) and alternation (Kolmogorov-Smirnov test, *P* > 0.14 for all cases; RM ANOVA test, prob. alternation: *F* = 0.16, *P* > 0.5) probabilities were similar across all SSIM values (Figures 3C,D).

We took a similar approach to explore the influence of stimulus discriminability on a [2AFC→ 2AUC] task for humans (Figure 3E). We tested two subgroups of participants using a counterbalanced experimental design (five participants each subgroup), with increasing (50% gray dots, left panels, Figure 3F) and decreasing (25% gray dots, center panels, Figure 3F; see also Supplementary Table S2) stimulus similarity (SIM). Both groups presented similar perceptual performances (RM ANOVA test, correct choices: *F* = 2.15, *P* = 0.11; *n* = 10), yet with some differences in their response times (RM ANOVA test, RT: *F* = 7.68, *P* < 0.001) (Trevino et al., 2013). Similar to our previous results with mice, despite the strong control that stimulus similarity exerted on visual performance, the participants showed similar choice bias (Kolmogorov–Smirnov test, *P* ≥ 0.13 for all cases; RM ANOVA test, prob. bias: *F* = 0.38, *P* = 0.98) and alternation (Kolmogorov–Smirnov test, *P* ≥ 0.12 for all cases; RM ANOVA test, prob. alternation: *F* = 0.88, *P* = 0.59) probabilities across all stimulus categories (upper and lower panels in Figures 3G,H). These results demonstrate that gradients in stimulus similarity did not change the production of choice biases in our [2AFC→ 2AUC] tasks for mice and humans.

### Dissociation Between Choice Biases and Stimulus Discriminability

**FIGURE 4:**
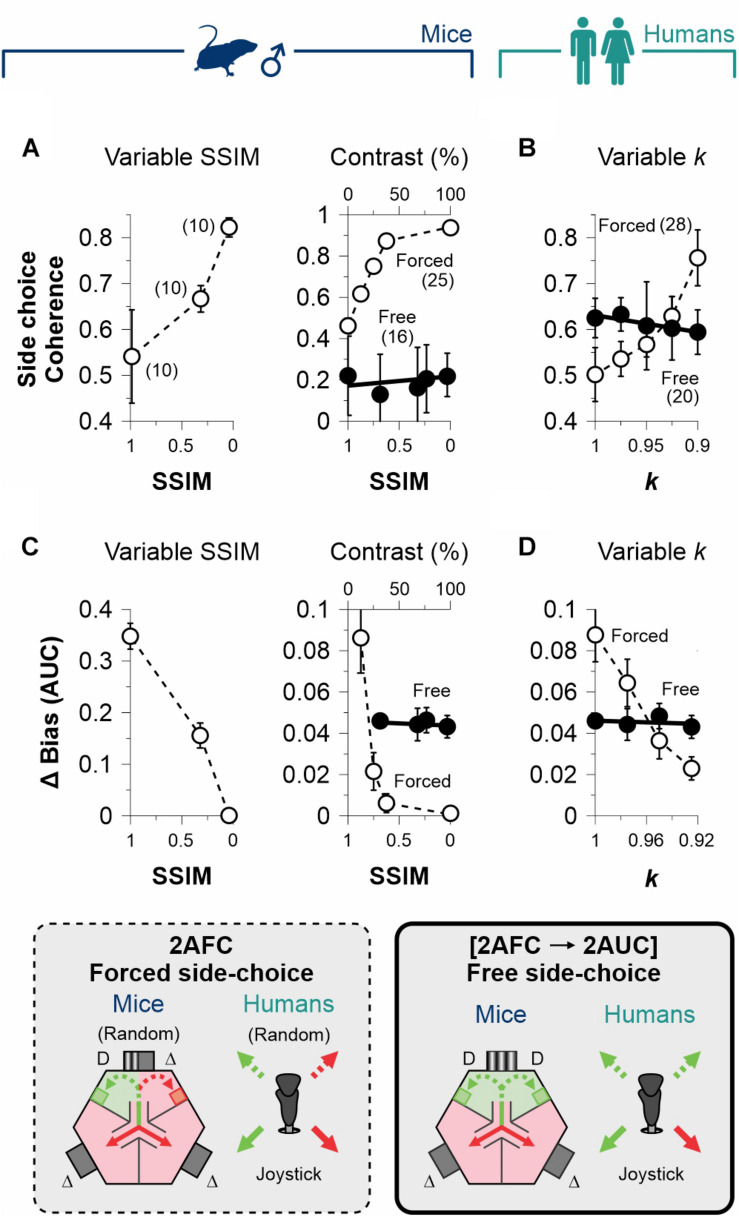
Dissociation of decisional biases from stimulus discriminability. **(A)** Group side-choice coherence as a function of stimulus discriminability in the 2AFC (empty circles) and 2AUC (filled circles) tasks for male mice (navy blue, **A**) and humans (dark cyan, **B**). Group coherence is sensitive to stimulus discriminability in the 2AFC tasks because subjects were tested with the same sequence of pseudo-randomized stimuli. Similarly, the difference in the area under the curve (AUC) of the bias probabilities reveals a dependency on discriminability for the 2AFC (empty circles) but not the 2AUC (filled circles) tasks for mice **(C)** and humans **(D)**. Thus, decisional biases in the 2AUC tasks are independent of stimulus discriminability. Number of subjects in parentheses.

In our 2AFC tasks, participants obtained reinforcement by perceiving and choosing an S^D^ with predictive value. This contingency established a direct link between stimulus discriminability and choice biases (Killeen et al., 2018). In contrast, stimulus discriminability did not affect the production of choice biases when we removed the predictive value of the S^D^ stimulus in the side-choice phase of the [2AFC→ 2AUC] tasks. These relationships can be visualized by graphing the group choice coherence against stimulus discriminability for mice (Figure 4A) and humans (Figure 4B). The rationale behind using the coherence metric is that all subjects were tested with the same sequence of pseudo-randomized stimuli. Accordingly, the group side-choice coherence increased with perceptual performance as a function of discriminability for mice (variable contrast | *F* = 7.98, *P* = 0.04, *n* = 25; empty circles, center panel, Figure 4A) and humans (*F* = 28.2, *P* = 0.01, *n* = 28; empty circles, Figure 4B) in the 2AFC task. Indeed, increased average performance in this condition involved making the same side-choices, because the locations of the stimuli among trials were equally distributed. However, no change in group coherence was observed in the experiments performed with the [2AFC→ 2AUC] paradigms (mice: *F* = 0.67, *P* = 0.47, *n* = 16; humans: *F* = 9.39, *P* = 0.07, *n* = 20; filled circles in Figures 4A,B). This is because mice and human participants could choose their preferred sides without any differential consequences in the [2AFC→ 2AUC] tasks.

An alternative way to confirm this finding: we calculated the area under the curve (AUC) of the changes in bias probabilities referenced to the one we obtained with max. discriminability (we refer to this measure as ΔBias). In agreement, stimulus discriminability had a strong influence on ΔBias in the 2AFC for mice (*F* = 27.45, *P* = 0.01; empty circles, Figure 4C) and humans (*F* = 114, *P* < 0.01; empty circles, Figure 4D), but this effect was virtually absent during the free-choice phase of the [2AFC→ 2AUC] tasks (mice: *F* = 1.03, *P* = 0.42; humans: *F* = 0.17, *P* = 0.72; filled circles, Figures 4C,D).

Our first experiment with the 2AFC task revealed how stimulus SSIM controlled decisional biases in mice. We wondered if a salient but non-predictive property of the sensory stimulus, for example, the drifting direction, could influence choice biases in mice. We trained a group of 10 mice in the [2AFC→ 2AUC] task with 100% contrast gratings drifting at 1 Hz to the right side. In these conditions, the probability of choosing the right side was much bigger than choosing the left one (Supplementary Figure S3B). In a second session, we trained the mice with right followed by left drifting gratings (33 trials/epoch). The probability of choosing the left or right sides revealed a dramatic change in preferred direction when comparing the same subjects during these two epochs (Kolmogorov–Smirnov test, *P* < 0.001 for both L_pre_/L_post_ and R_pre_/R_post_ comparisons; RM ANOVA test, prob. Bias L_pre_/L_post_: *F* = 10.17, *P* < 0.001; RM ANOVA test, prob. Bias R_pre_/R_post_: *F* = 14.21, *P* < 0.001; Supplementary Figure S3C). These results imply that salient attributes of the sensory stimuli can influence choice biases, even when they are not predictive in the [2AFC→ 2AUC] task.

**FIGURE 5:**
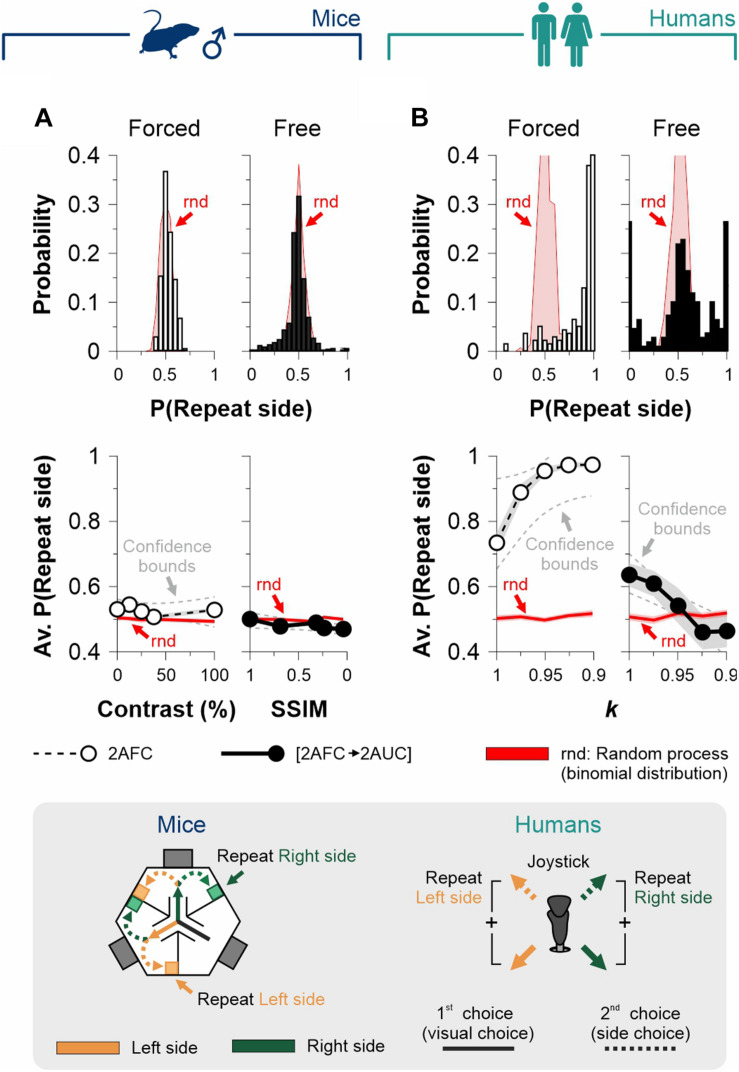
Assessment of sequential effects in the 2AFC and [2AFC→ 2AUC] tasks for mice and humans. We calculated and extracted the probabilities that side-choices involved the same side as the previous visual choice during each trial (P[repeat side]) for the 2AFC tasks (empty bars and circles) and the [2AFC**→** 2AUC] tasks (filled bars and circles) for male mice **(A)** and human participants **(B)**. Upper panels show the probability distributions, whereas the lower panels show the average probabilities as a function of stimulus discriminability. Red distributions and traces correspond to a random process derived from a binomial distribution involving the same number of observed trials. Note how, for mouse tasks, the P(repeat side) were similar to chance distributions. This implies that arm selection and side choice were fully independent processes. In contrast, in the human tasks, the P(repeat side) shows considerable differences against chance distributions (upper panels), which is also reflected as a dependence of P(repeat side) on stimulus discriminability (lower panels).

Because choice probabilities are not only sensitive to the current stimulus strength but also to the history of preceding events, we explored for sequential effects in the choices of mice and human participants in our 2AFC and [2AFC→ 2AUC] tasks. More specifically, we calculated the probability that the side-choice involved the same side as the one found with the previous visual choice during the same trial (P[repeat side]). We found that both mouse task variants lacked such sequential effects (empty bars, 2AFC: av. P[repeat side] = 52.7% ± 3.6%, independent of stimulus contrast, *F* = 0.136, *P* = 0.73; filled bars, [2AFC→2AUC]: av. P[repeat side] = 48.2% ± 3.8%, *F* = 7.4, *P* = 0.06; Figure 5A), whereas the side choices for both human tasks exhibited a non-random influence of previous visual choices (empty bars, 2AFC: av. P[repeat side] = 90.5% ± 1.1%, independent of stimulus contrast, *F* = 17.99, *P* = 0.01; filled bars, [2AFC→2AUC]: av. P[repeat side] = 54.3% ± 2.2%, independent of stimulus contrast, *F* = 40.01, *P* < 0.001; Figure 5B). Therefore, the mouse [2AFC**→** 2AUC] task lacks sequential effects, making it an idoneous tool to study discriminative capacities and choice biases separately. The sequential effects found in both human tasks constitute an important influence to consider when studying human side choices.

### Stable Production of Stereotyped Choices Across Different Days

**FIGURE 6:**
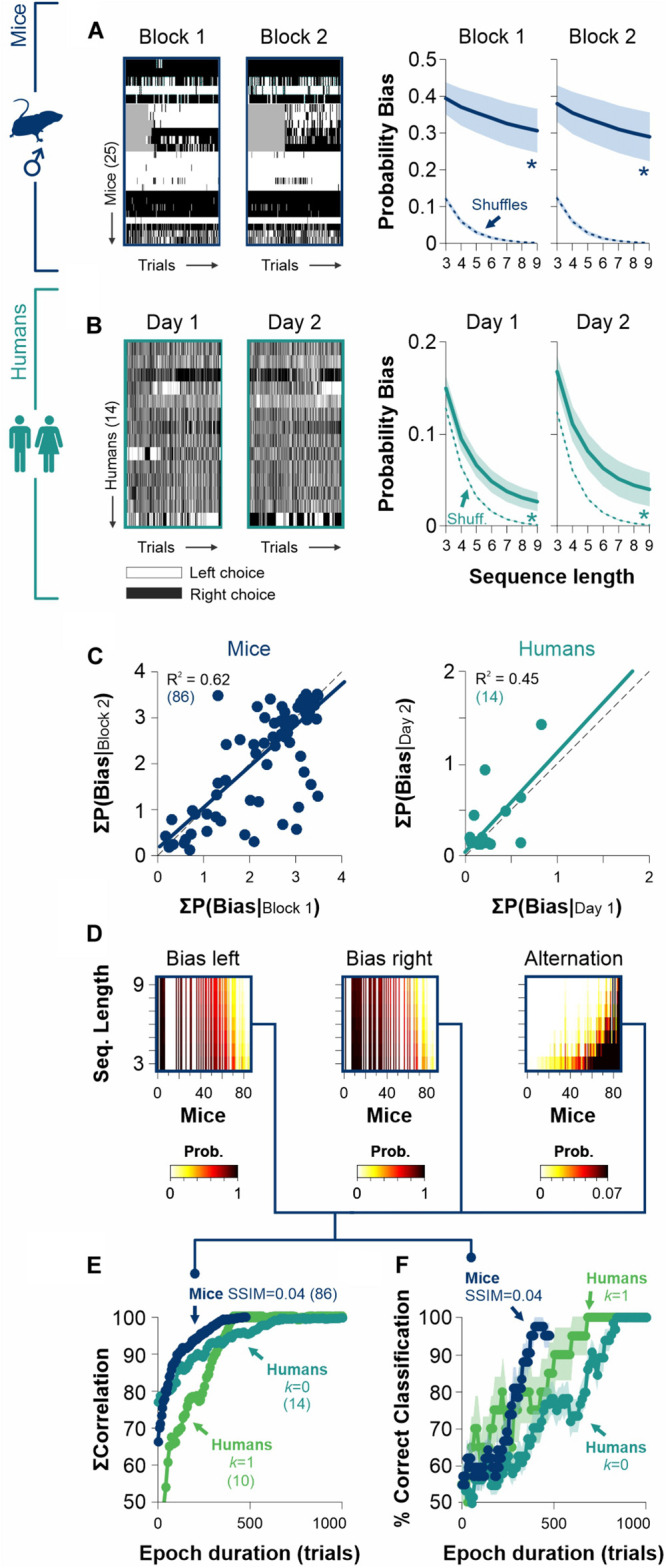
The stability of biased and alternating side-choice behavior serves to identify participants. **(A)** Side-choice colormaps (left) and side-choice probabilities for male mice (navy blue) measured in two different epochs with a gap of 146 days between them. **(B)** Side choices of humans (dark cyan) are also relatively stable across different days. Side-choice colormaps on the left. **(C)** Scatter plots of the area under the curve (AUC) for the bias probabilities obtained in pre. vs. post. epochs for mice (left panel) and humans (right panel). **(D)** Sample side-choice bias/alternating probabilities extracted from 500 trials of 86 male mice solving the 2AUC task with high discriminability (SSIM = 0.04). **(E)** Cumulative match characteristic curves as a function of epoch duration. We made comparisons with the summed correlations (left, right, and alternating probabilities) as a function of epoch duration. **(F)** The same bias/alternating probabilities extracted from epochs of different lengths can be used to identify the mice (navy blue) and human participants (parakeet green, zero discriminability: *k* = 1; dark cyan, high discriminability: *k* = 0) with increasing accuracies for longer epochs. Number of subjects in parentheses.

The existence of free choice biases that are relatively constant across experimental sessions can be easily explained in terms of a biased decision rule. Furthermore, choice biases that are robust and independent of stimulus discriminability favor the notion that they should derive from a stable internal representation. We explored the choice data from our [2AFC→ 2AUC] experiments and found that mice had strikingly similar choice biases when measured across different epochs (w. a gap of 146 days between testing epochs, RM ANOVA test, Probability Bias| mice: *F* = 0.31, *P* = 0.86; paired t-test: *P* = 0.76, *n* = 25, Figure 6A). Humans also showed remarkably similar choice biases along consecutive days (RM ANOVA test, Probability Bias| humans [*k* = 0]: *F* = 1.30, *P* = 0.27; paired *t*-test: *P* = 0.11, *n* = 14, Figure 6B). The intra-individual consistency and replicability in the production of different degrees of stereotyped choosing can be further appreciated with a scatter plot which compares the summed bias probability distributions for mice and humans across different epochs (Figure 6C). Indeed, the linear regressions had strong coefficients of determination (mice data in navy blue: *R*^2^ = 0.96, *F* = 142, *P* ≤ 0.001; human data in dark cyan: *R*^2^ = 0.45, *F* = 17.5, *P* ≤ 0.001; Figure 6C). Similarly, the probability of alternations was quite stable across epochs for both groups (Probability Alternation| mice: *F* = 0.76, *P* = 0.54, R^2^ = 0.73, *F* = 69.2, *P* ≤ 0.001; Probability Alternation| humans [*k* = 0]: *F* = 0.31, *P* = 0.86, *R*^2^ = 0.09, *F* = 2.28, *P* = 0.15; Supplementary Figure S4). These results demonstrate the longitudinal robustness of choice biases in mice and humans.

Because choice bias probabilities are quite invariant in time, this opens the door to use them as a behavioral feature to identify individuals. Using choice records from 500 trials from a group of 86 mice, we calculated and sorted the probability distributions for biased left and right choices and complementary alternating sequences (i.e., ‘LR…L’ + ‘RL…R’; Figure 6D). Next, we computed the cumulative match characteristic curves with increasing epoch durations. In Figure 6E, we illustrate these comparisons for the summed correlations as a function of epoch duration (i.e., the number of cumulative trials from which probabilities were estimated; navy blue for mice). We also repeated this analysis for human participants tested in low and high discriminability conditions (parakeet green for humans with *k* = 1, and dark cyan for humans with *k* = 0). Note how the cumulative match characteristic curves increased rapidly with the number of trials considered.

Biometric systems identify participants by using physiological and behavioral predictors. To further explore the possibility of using choice biases to identify individual mice/participants, we implemented a simple linear classifier to identify each mouse/participant by minimizing the Euclidean distance between observed and final biased and alternating probabilities. As expected, the accuracy of the classifier grew by increasing the epoch duration (Figure 6F). Interestingly, the classification accuracy improved faster for mice than for humans, reflecting that mice are easier to identify through their choice biases.

### Adaptive Production of Choice Biases Through Reinforcement

Choice biases constitute an unvarying repetition of responses toward a particular side. This may, or may not, serve a particular function. Mice are well known to adapt their search strategies depending on the experimental context. For example, in navigation studies, mice initially solve tasks using a place-learning navigation strategy, but subsequently, they learn to use a response-based navigation strategy (Trevino et al., 2013; Trevino, 2014). Well-learned sequences tend to become automatic; they are performed faster and require less attention than with new tasks. Nevertheless, variability can also be useful for exploration, and it can be intentionally increased to search for actions that may yield more reinforcement.

**FIGURE 7:**
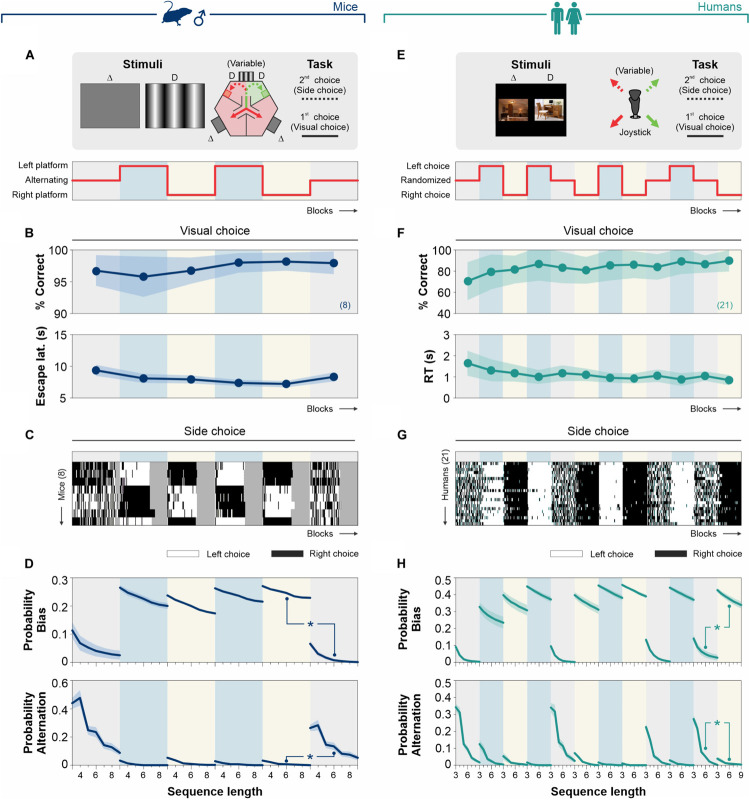
Reinforcement of biased and alternating choices. Task structure with a volatile (alternating reinforcement) or with a stationary (left or right reinforcement) rewarding landscape for mice (navy blue, **A**) and humans (dark cyan, **E**). **(B)**% Correct choice and escape latencies of the mice obtained with a 100% contrast stimulus along with blocks of trials with alternating (10% gray background), left (sky blue), or right (lemon yellow) reinforcement. **(C)** Colormaps of side-choices of individual male mice, with black (right choices) or white (left choices) rectangles across experimental manipulations. This experiment involved six epochs of 520 trials acquired along 12 days/epoch. **(D)** Probability of occurrence for biased (upper row) and alternating (lower row) probabilities as a function of sequence length for the different experimental blocks. **(F)**% Correct choices and response times obtained with stimuli with high discriminability (i.e., *k* = 0) along with blocks with balanced randomized (10% gray background), left (sky blue), or right (lemon yellow) reinforcement. **(G)** Side-choice colormaps for human participants, along with the different training blocks. **(H)** Probabilities for biased (upper row) and alternating (lower row) choices. Number of subjects in parentheses.

In 2AFC tasks, the reinforced locations are randomized so that side-choice biases lead to random performance (Herrera and Trevino, 2015; Abrahamyan et al., 2016). As a fundamental mechanism of trial-and-error learning, decision-makers can adjust their motor variability to improve task performance, particularly in tasks with uncertain conditions (Dhawale et al., 2017). Furthermore, some reports indicate that human participants can quickly adapt to operant contingencies that specify different levels of variability, such that different degrees of repetitive behaviors can be produced ‘on demand’. In other words, repeating rewards on the same location for multiple trials creates an imbalance in reinforcement that favors one side over the other (Neuringer, 2002; Herrera and Trevino, 2015). Taking this into account, we hypothesized that mice and humans should change their side-choice behavior in response to targeted imbalances in the side of reinforcement. To test this idea, we employed stimuli with high discriminability (for mice: a grating stimulus with 100% contrast and 0.04 cycles/degree; for humans: two images with *k* = 0, see section “Materials and Methods”) and trained the mice/participants across different training blocks with stationary or variable reward landscapes, by reinforcing (i) alternating, (ii) left, or (iii) right sequences (Figures 7A,E).

The discriminative choices of a group of eight mice were stable across six experimental blocks (average performance/block), with a subtle reduction in their escape latencies over training (non-Parametric Kruskal–Wallis test with Bonferroni correction for% correct choices: *F* = 2.69, *P* = 0.75; for escape latencies: *F* = 10.76, *P* = 0.06; Figure 7B and Supplementary Table S3). The probability of producing side choices was high and similar across days in which we reinforced left-only (white rectangles) or right-only (black rectangles) sides (Kolmogorov–Smirnoff test, *P* ≥ 0.23 for all cases, Figures 7C,D). Indeed, this probability was ∼163% higher (AUC) than when reinforcing alternation (i.e., placing the reinforcement in alternating sides during the training blocks; KS test, *P* < 0.001 all cases; upper panels, Figure 7D). Similarly, the probability of producing alternating sequences was similar across days in which we reinforced left/right alternations (Kolmogorov-Smirnoff test, *P* ≥ 0.70 for all cases), and it was >2000% bigger compared to those blocks in which we reinforced side-choices (KS test, *P* < 0.001 all cases; lower panels, Figure 7D).

We conducted a similar experiment in 21 human participants across 12 experimental blocks (Figure 7F and Supplementary Table S3). There was a minor increase in perceptual performance (average/block) between day 1 (*d*_1_) and [*d*_10_, *d*_12_] (KW-test with *post hoc* Bonferroni correction, *F* = 63.10, *P* < 0.05) and a reduction in RTs when comparing *d*_1_ and [*d*_8_, *d*_10_, *d*_12_] (KW-test, *F* = 54.92, *P* < 0.05). Similar to what we found in mice, the biased probability was similar across days during which we reinforced side-choices (Kolmogorov–Smirnoff test, *P* ≥ 0.31 for all cases), but it was ∼970% bigger (AUC) than the biased probability obtained when reinforcing randomized choices (KS test, *P* < 0.001 for all cases; upper panels, Figure 7H). Alternation probabilities were similar across days in which we reinforced alternation (Kolmogorov-Smirnoff test, *P* ≥ 0.47 for all cases) but were ∼797% bigger than the alternation probability we found when reinforcing side-choices (KS test, *P* < 0.001 for all cases; lower panels, Figure 7H). These results demonstrate that the production of choice biases in our tasks adapts to recent reward history and that different levels of choice stereotypy can be reinforced in mice and humans.

To further explore the sensitivity of mouse side-choice behavior to external influences, we reinforced the un-preferred side of a group of 8 mice for two consecutive days, while measuring side-choice behavior before (Pre), during (reinforcement), and after (Post) this training procedure. The asymmetric reinforcement had clear effects in the observed biased choices (azure blue, Trained vs. Pre: *m* = 0.03, *R*^2^ = 0.03, *F* = 0.22, *P* = 0.65, *n* = 8) but, remarkably, the general profile of side-choice behavior of these mice quickly returned to baseline values after concluding reinforcement (navy blue, Post vs. Pre: *m* = 0.79, *R*^2^ = 0.386, *F* = 111, *P* < 0.001; Supplementary Figure S5). We found a similar phenomenon with the alternating choices (Trained vs. Pre: m = 0.02, *R*^2^ = 0.03, *F* = 0.21, *P* = 0.66; Post vs. Pre: m = 0.75, *R*^2^ = 0.45, *F* = 11.7, *P* < 0.004). These results suggest that adjustments in side-choice behavior can be reinforced, but the idiosyncratic side-preferences of the mice tend to return to a baseline level once the differential reinforcement is removed.

### Discriminative, Reinforcement, and Choice-History Factors Influence the Production of Biased Choices

**FIGURE 8:**
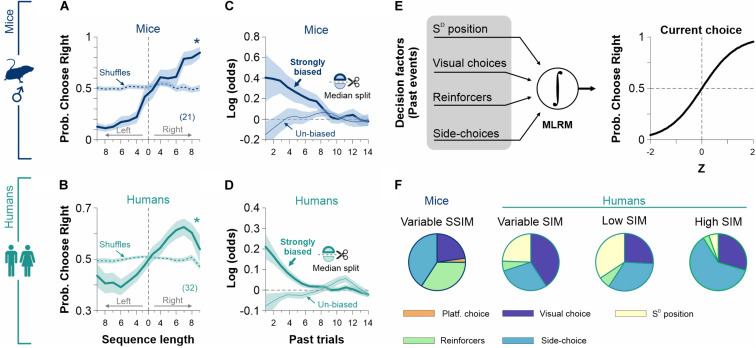
Past choices and reinforcers influence decisional biases. The probability of choosing a particular side (left or right) is strongly influenced by the amount of previous side-biased choices for male mice (navy blue, **A**) and humans (dark cyan, **B**). The weighted coefficients from a multiple linear regression model (MLRM) confirm a strong dependency on past trials for strongly biased mice (thick navy blue line, **C**) and humans (thick dark cyan line, **D**). **(E)** We incorporated (i) past choices, (ii) reinforcers, (iii) side-choices, and (iv) S^D^ position to the MLRM. **(F)** Side-choices from the experimental groups displayed different sensitivities to the four predictors depending on how stimulus discriminability was varied during training. These results support the notion that the production of side-choices is highly adaptable (Trevino et al., 2013). Number of subjects in parentheses.

Choices are not determined exclusively by current sensory information; they are also influenced by past experiences, decisions, and outcomes (Linares et al., 2019). Indeed, animals guide their choices based on the outcomes of past decisions (Abrahamyan et al., 2016). Moreover, depending on the particular experimental context and the amount of information provided, mice and humans can adopt different task-solving strategies. Such strategies can include repeating/avoiding previously rewarded/un-rewarded choices, alternating choices, or more complex combinations (Corrado et al., 2005; Lau and Glimcher, 2005; Busse et al., 2011; Jones et al., 2013; Trevino, 2014; Abrahamyan et al., 2016; Killeen et al., 2018; Chen et al., 2019; Linares et al., 2019). To explore how previous side-choice biases influenced current decisions, we first implemented an event-triggered average procedure to estimate the probability of preferring a particular side after executing side sequences. We found that the likelihood of choosing a particular side (left or right) increased with the length of the previously executed biased sequence both for mice (Figure 8A) and humans (Figure 8B). To corroborate this observation, we implemented a multiple logistic regression model (MLRM) to quantify the dependence of current biased choices on past choices and reinforcers (see section “Materials and Methods”; Figures 8C,D). The coefficients of the MLRM were calculated using 20 previous trials, and their statistical significance was tested using a permutation test with 1,000 shuffles (Trevino, 2014). Furthermore, we used a median split to separate strongly and poorly biased subgroups of mice and humans. Positive coefficients in the strongly biased cases (thick lines) reflect that biased choices increased the probability of repeating the same side on the next trial. In contrast, negative coefficients in the un-biased cases (thin lines) indicate alternation on the subsequent trial (Figures 8C,D). The decaying effect of past choices and reinforcers reveals that 6 and 10 past trials influenced current choice for mice and humans, respectively (Figures 8C,D) (Abrahamyan et al., 2016).

Adapting another MLRM, we next explored how past (i) S^D^ positions, (ii) visual choices, (iii) reinforcers, and (iv) side-choices influenced the production of current side-choices from our mice and human experiments (Figure 8E). We hypothesized that the exact contribution of these factors could vary with the particular discriminability trajectories that we used during our experiments (Trevino et al., 2013). In agreement, we found that the contribution of these predictors was specific for each experimental condition (pie charts were built using the summed MLRM coefficients using a history of 6 past trials; Figure 8F). Altogether, these results illustrate how previous choice biases strongly influence side-choices. Both mice and humans updated their strategies and adapted their side-choices to cope with the particular demands of the experiments.

## Discussion

We adapted two-alternative tasks for mice and humans to study their visual capacities and choice biases (Trevino et al., 2018). The tasks were easy to use, allowing us to test ≥1,300 trials/day for a group of 20 mice (Trevino et al., 2018) and 1,000 trials/day (Trevino et al., 2016) for each human participant. Through a forced-choice task (2AFC), we measured detection and discrimination performance, whereas an extension involved coupling the 2AFC task with a free-choice (2AUC) task. The combination of both tasks ([2AFC→2AUC]) in a single trial allowed us to explore discrimination and choice biases simultaneously. We defined such ‘choice biases’ simply as the tendency to choose more one alternative over another.

Our results offered a side-by-side comparison of rodent and human findings. By adjusting the similarity of training stimuli, we found that stimulus discriminability reduced choice biases in the 2AFC tasks for both species. In the [2AFC→ 2AUC] task, the stimulus predicted reinforcement during the forced-choice (visual choice) but was irrelevant during the second free-choice (left or right side-choice), as both sides were equally reinforced. In consequence, the production of choice biases in the [2AFC→2AUC] tasks was insensitive to changes in stimulus similarity. Thus, stimulus discriminability had a direct influence on the production of choice biases in forced-choice but not in free-choice tasks. Using the [2AFC→ 2AUC] tasks, we found that choice biases varied substantially in magnitude and preferred side across individuals (Abrahamyan et al., 2016; Urai et al., 2019). The mixed side preferences imply that the choice biases were not a consequence of asymmetries in the experimental apparatus (Trevino, 2014). Furthermore, we found that individuals’ stereotypical choice behavior (mice and humans) was strikingly stable in appearance and intensity across experimental days, which is difficult to explain in terms of dysfunction.

We explored for sequential effects between visual and side-choices. Only for the mouse tasks, we found that the side choices were fully independent of the visual choices. In contrast, the side-choices in the [2AFC→2AUC] task for humans carried some information from the previous discriminative decisions. Therefore, the lack of sequential effects in the mouse [2AFC→2AUC] task makes it an ideal experimental platform to explore the non-sensory contributions and mechanisms involved in side-choice behavior. Furthermore, the side-choices of the mice also exhibited sensitivity to salient features (drifting direction) of non-predictive stimuli.

One limitation of this study is that we only used male mice, and we did not explore gender differences. Although male and female mice usually reach similar performance levels, they tend to adopt different strategies during learning, with male mice changing strategies more frequently than females (Chen et al., 2019). Despite the existence of such potentially exciting differences, we did not include them as a primary aim of our study, mainly because studying them would require simultaneously measuring hormone levels and side-choice behavior during different phases of the estrous cycles of the females. This technically challenging question remains open for a follow-up study.

Side choices exist in 2AFC tasks despite producing sub-optimal performance (Trevino, 2014; Akrami et al., 2018). They could derive from the uncertain nature of decisions themselves together with learned strategies about which side predicts reward based on past experiences with the task (Killeen et al., 2018; Chen et al., 2019). However, side-choice behavior could also involve some innate preferences that would go beyond establishing responses to sensory stimuli. Indeed, many behaviors, including responses to visual stimuli, are largely innate (Zador, 2019). In our experiments, we found that side-choice behavior strongly persisted after removing the dependency of the side choices on the discriminative stimulus in the [2AFC→2AUC] task for mice. This property reflects that side-choice behavior derives from a stable internal representation that lasted many months for our mice. Maybe not surprisingly, the propensity to develop motor stereotypies depends on genomic factors (Peter et al., 2017). Some innate behaviors, such as exploring or avoiding predators, could be sculpted by evolution into stereotyped modules that encode coherent and adaptive patterns of action (Wiltschko et al., 2015; Abrahamyan et al., 2016). Many fixed behavioral patterns are essential for survival, and highly skilled acts involve multiple repetitive actions (Langen et al., 2011). Thus, some decisional stereotypies might involve an innate component since there are many long-term regularities in nature (Akrami et al., 2018). Other stereotypies could reflect reinforced habits that emerge through development and become persistent and relatively hard to abolish (Langen et al., 2011). Our experiments demonstrate that choice biases are adaptable. From this perspective, stereotypies could emerge and be sustained by their rewarding consequences. The ‘coping hypothesis’ considers that stereotypic behavior is adaptive and is based on purely motivational processes.

In stable rewarding landscapes, improving performance usually means decreasing trial-to-trial variability with practice (Dhawale et al., 2017). However, when task conditions are more uncertain, trial-and-error learning constitutes a powerful strategy to optimize solutions (Abrahamyan et al., 2016). In our experiments, we found that side and alternating choice sequences could be reinforced both in mice and humans. Evidence from other groups suggests that controlled variability in motor output could be beneficial for increasing reinforcement (Wu et al., 2014; Pekny et al., 2015). Thus, task-relevant variability could provide a unifying explanation for individual differences in learning rates across tasks (Wu et al., 2014). Besides, stereotyped modules of behavior exhibit moment-to-moment variability in such a way that intra-individual variability could itself be a signature of motor development (Wiltschko et al., 2015). In humans, intra-individual variability decreases rapidly in the first few months after walking onset, followed by a long period of gradual improvements to support skilled performance in which consistency finally approximates that of adults.

A source for choice biases and their dependence on recent history could involve stable working memory representations (Fischer and Whitney, 2014; Fritsche et al., 2017; Urai et al., 2019). History effects on choice have been observed in humans (Fischer and Whitney, 2014; Abrahamyan et al., 2016) and rodents (Busse et al., 2011; Akrami et al., 2018) and are present in forced-choice (Gold et al., 2008; Fischer and Whitney, 2014), memory-guided (Akrami et al., 2018; Hermoso-Mendizabal et al., 2018), and free-choice tasks (Sugrue et al., 2004; Lau and Glimcher, 2005). We also found that mice and humans adapted their behavioral strategies depending on the discriminability trajectories established during training (Ahissar et al., 2009; Trevino et al., 2013). From these and other experiments, it is clear that experience plays a vital role, especially when the external conditions are uncertain (Trevino, 2014; Abrahamyan et al., 2016). These adaptations could reflect a Bayesian inference strategy, where the influence of previous choice biases is more substantial when sensory evidence is scarce. In other words, such behavior could rely on subjects tracking the value of the probability to obtain reward following each choice, an adaptive strategy when these probabilities change slowly in time (Corrado et al., 2005; Lau and Glimcher, 2005). In sum, choice biases were robust and relatively stable, but they were also adaptable, allowing both mice and humans to update their task-solving strategies to cope with the particular demands of the experiments. Interestingly, the experiments included reinforced side-choices and involved reward contingencies that were reversed across training blocks. Reversal learning is a common paradigm to measure the ability to suppress previously rewarded responses and disengagement from ongoing behavior. For this reason, reversal learning is thought to change the flexibility of responses, and it may be informative of impulsive and compulsive behaviors in a variety of psychopathologies (Izquierdo and Jentsch, 2012). It would be interesting to explore whether and how the strength of side-choice biases in mice and humans influence the rates of reversal learning.

Low levels of behavioral variability and/or high stereotypy characterize some human psychopathologies and mental disorders (Neuringer, 2002). Human stereotypies are prevalent in autism spectrum disorders (ASD), stereotyped movement disorders, and a range of other syndromes that involve some degree of intellectual disability (Langen et al., 2011). One possibility is that the stereotyped behavior seen in autistic children could be a secondary consequence due to abnormal information processing. Recent studies suggest excessive neural variability and abnormal synchronization of neural activity across distant brain areas in individuals with autism (Dinstein et al., 2015). Stereotypies are also common in schizophrenia, Tourette’s syndrome, and in some obsessive-compulsive disorder patients (Frith and Done, 1983). In contrast, other psychopathologies, such as attention deficit hyperactivity disorder (ADHD) and Down syndrome, show elevated amounts of intraindividual variability in basic motor skills (Neuringer, 2002).

At the circuit level, we still do not understand the mechanisms that underlie choice biases and why these traits are present in different proportions in mice and humans. Irrespective of whether side choice behavior is innate or learned, the fact that we could use it to identify subjects clearly indicates that this behavior should have a stable internal representation, one which could derive from functional asymmetries in neuronal circuitry. Internal factors and asymmetries in sensory encoding and inhibitory control have been associated with choice biases (Langen et al., 2011; Novak et al., 2016; Jin and Glickfeld, 2019; Linares et al., 2019). Most importantly, stereotypies in mice could share some mechanisms with stereotypies in human mental disorders (Langen et al., 2011). Thus, developing quantitative tools to measure stereotypical choice behavior in mice opens the possibility to study the participation of different brain regions, particularly with unilateral and/or bilateral circuit inactivations (Trevino et al., 2018).

Variability at the circuit level is generated by many neurophysiological mechanisms that include the stochastic nature of synaptic transmission with relevant interactions across large neural populations and with distributed neuromodulation effects (Dinstein et al., 2015; Trevino et al., 2019). Where exactly does the brain implement regulation for choice variability? The circuits implementing such variability should have information about past performance and should have the capacity to influence motor output. Impaired basal ganglia function and imbalances in corticostriatal function have been linked to some forms of repetitive behavior (Frith and Done, 1983; Langen et al., 2011). The posterior parietal cortex (PPC) has also been found to participate in the production of decisional biases with a strong history dependence (Wilke et al., 2012; Akrami et al., 2018). At the genetic level, the hyperdopaminergic DAT knock-out mouse exhibits ‘superstereotypies’ which involve a series of fixed action patterns (Langen et al., 2011). The disruption of the Shank3 gene in mice (a mutation found in some cases of autism and intellectual disability) is linked to alterations in glutamatergic synapses and autistic-like behaviors (Wang et al., 2011; Berkel et al., 2012).

There is a lack of knowledge of the mechanisms that mediate and regulate the manifestation of repetitive behaviors (Peter et al., 2017). Analysis of choice stereotypies could be used as a tool for diagnosis for psychopathologies that involve this type of behavior. We consider it essential to distinguish the external factors that influence a contingent repetition of learned behaviors, from internal representations that underlie well established and purposeless stereotypies. At the treatment level, excessive stereotypical behavior found in many psychopathologies may be reduced or eliminated through differential reinforcement (Neuringer, 2002). Behavioral therapies focused on habit reversal and differential reinforcement could become useful to treat motor stereotypies.

## Data Availability Statement

The raw data supporting the conclusions of this article will be made available by the authors, without undue reservation.

## Ethics Statement

The studies involving human participants were reviewed and approved by ET092018-271; Instituto de Neurociencias, Universidad de Guadalajara, Mexico. The participants provided their written informed consent to participate in this study.

## Author Contributions

MT conceived the project, designed and built the devices, made projection and analysis algorithms, analyzed the data, made the figures, wrote the manuscript, and answered the reviewers’ comments. RM-C adjusted the projection of visual stimuli, oversaw the proper functioning of devices used for behavioral experiments, and made figures. BH performed the tests with human participants.

## Conflict of Interest

The authors declare that the research was conducted in the absence of any commercial or financial relationships that could be construed as a potential conflict of interest.

## Supplementary Material

FIGURE S1 Visual discrimination tasks for mice and humans with variable discriminability. **(A)** Scheme of our previous 2AFC task for mice: two monitors facing the ends of the arms of a Y-maze display the discriminative (S^D^, reinforced) and delta (S^D^, non-reinforced) stimuli (100% contrast). A submerged transparent platform below the S^D^ serves as the unconditioned stimulus (US). The position of both the platform and S^D^ in either arm varies pseudo-randomly over consecutive trials. **(B)** Average correct choices (after 275 training trials) as a function of stimulus discriminability. **(C)** Side-choice colormaps for the male mice (white: left choices, black: right choices). **(D)** Side-choice and alternation probabilities are strongly modulated by stimulus discriminability (Trevino, 2014). **(E)** Psychometric curves from the 2AFC task for humans depicting the % Correct choices as a function of *k* (*x*-axis; the scalar with which we made the linear combination of images to create S^D^ and S^D^ stimuli). Each participant is represented with a different line color. Panels below show the average response times (RT) as a function of *k*. Responses were faster in easier conditions. Number of subjects in parentheses.

FIGURE S2 Discriminability reduces decisional biases in 2AFC tasks. The area under the curve (AUC) of the changes in bias probabilities referenced to the one we obtained at max. discriminability (i.e., the ΔBias) drops as a function of stimulus discriminability for mice (continuous navy blue line, **A**) and humans (continuous dark cyan line, **B**). In contrast, the alternation probabilities increased with stimulus discriminability for both groups (dotted lines). Number of subjects in parentheses.

FIGURE S3 Decisional biases are influenced by drifting direction in a [2AFC→ 2AUC] task for mice. **(A)** Cartoon of the [2AFC→ 2AUC] task for mice in which the discriminative stimulus consisted of a drifting sine-wave grating. **(B)** Choice results from the task with the first block of training with a 1 Hz drifting grating to the right side. The second and third panels display the left and right side-choice probabilities. **(C)** On a second experiment with the same mice, we trained them with a right followed by left drifting gratings. Probability distributions on the second and third panels. Number of subjects in parentheses.

FIGURE S4 Stable alternating choice probabilities in the 2AUC task for mice and humans. Stable alternating choices for male mice **(A)** and humans **(C)** in conditions of high discriminability across 2 days/epochs. We used 1,000 shuffles to determine the statistical significance of the observed probabilities (dotted lines). Linear regressions comparing the summed probabilities of alternating choices for mice (navy blue, **B**) and humans (dark cyan, **D**) across days/epochs.

FIGURE S5 Challenging the stability of side-choice behavior. Comparisons of side (left panel) and alternating (right panel) choice behavior during (azure blue dots, *m* = 0.03) or after (navy blue dots, *m* = 0.79) reinforcing the un-preferred side of 8 mice during two days of training. A slope close to one reflects that changes in side-choice behavior by reinforcement vanished quickly after training finished.

FIGURE S6 Predictors of decisional biases. Sample predictors used as input data for the multiple linear regression model (MLRM, see section “Materials and Methods”) used to predict choices from mice **(A)** and humans **(B)**. Note how the group coherence of visual choices increases with stimulus discriminability. Number of subjects in parentheses.

TABLE S1 Average performance of mice and humans in the 2AFC tasks. This table corresponds to the empirical data (average ± SEM) we illustrate in panels from Figures 2B,F.

TABLE S2 Average performance of mice and humans in [2AFC→ 2AUC] tasks. This table corresponds to the data (average ± SEM) we illustrate in panels from Figures 3B,F.

TABLE S3 Average performance of mice and humans while reinforcing side-biased and alternating strategies. This table corresponds to the analyzed data (average ± SEM) we illustrate in panels from Figures 7B,F.
