# Supplementary material for: Adaptive Choice Biases in Mice and Humans
Source: Front Behav Neurosci. 2020 Jul 14;14:99. doi: 10.3389/fnbeh.2020.00099 (PMC7372118; doi:10.3389/fnbeh.2020.00099)
Supplement: FIGURE S1 — Visual discrimination tasks for mice and humans with variable discriminability. (A) Scheme of our previous 2AFC task for mice: two monitors facing the ends of the arms of a Y-maze display the discriminative (SD, reinforced) and delta (SD, non-reinforced) stimuli (100% contrast). A submerged transparent platform below the SD serves as the unconditioned stimulus (US). The position of both the platform and SD in either arm varies pseudo-randomly over consecutive trials. (B) Average correct choices (after 275 training trials) as a function of stimulus discriminability. (C) Side-choice colormaps for the male mice (white: left choices, black: right choices). (D) Side-choice and alternation probabilities are strongly modulated by stimulus discriminability (Trevino, 2014). (E) Psychometric curves from the 2AFC task for humans depicting the % Correct choices as a function of k (x-axis; the scalar with which we made the linear combination of images to create SD and SD stimuli). Each participant is represented with a different line color. Panels below show the average response times (RT) as a function of k. Responses were faster in easier conditions. Number of subjects in parentheses. [file Image_1.pdf]

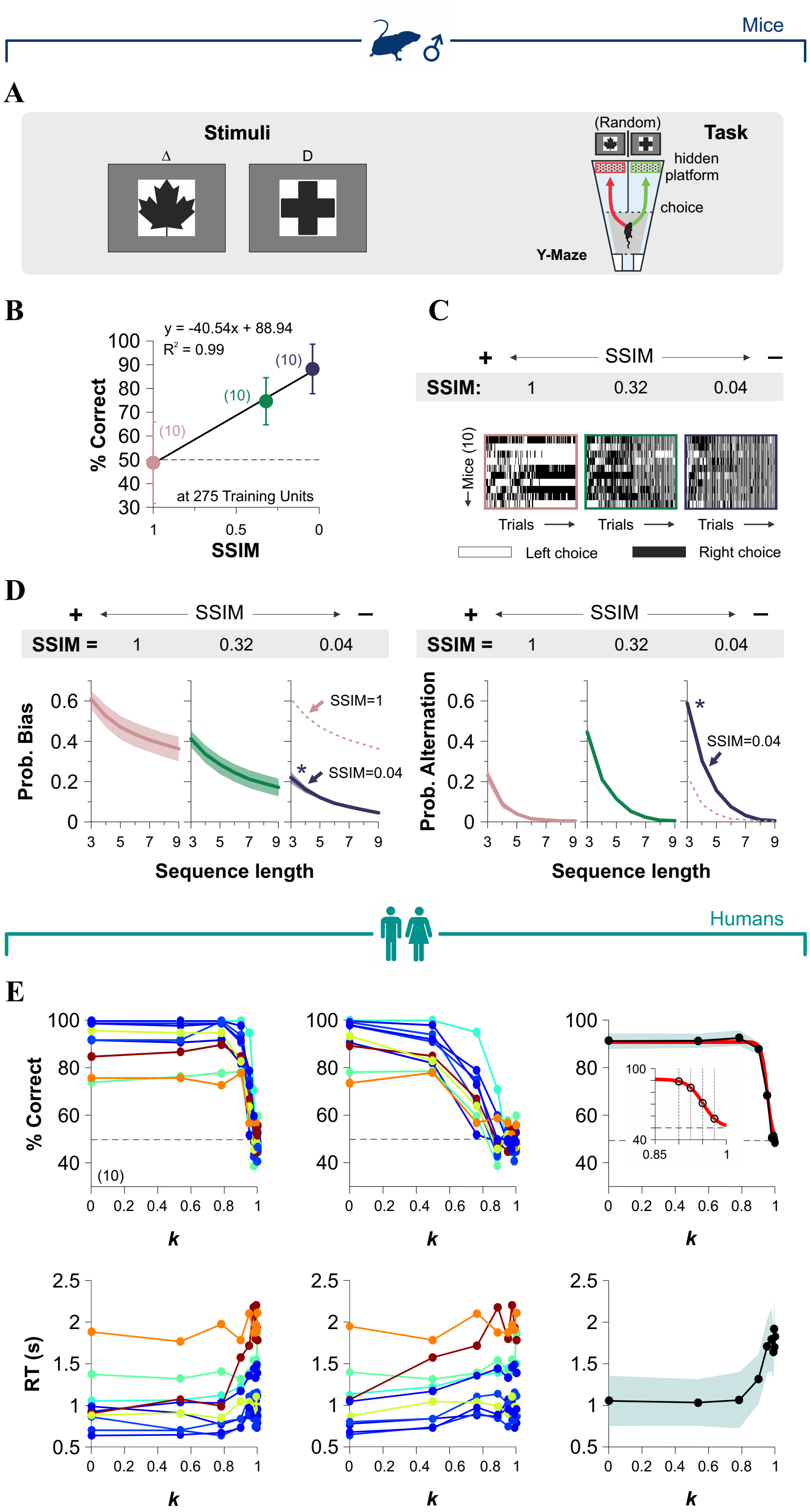

Supplementary Figure 1

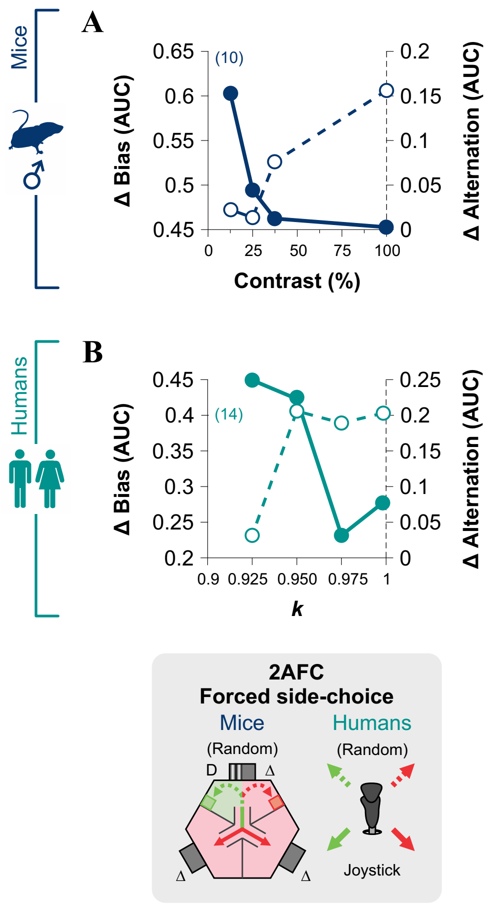

Supplementary Figure 2

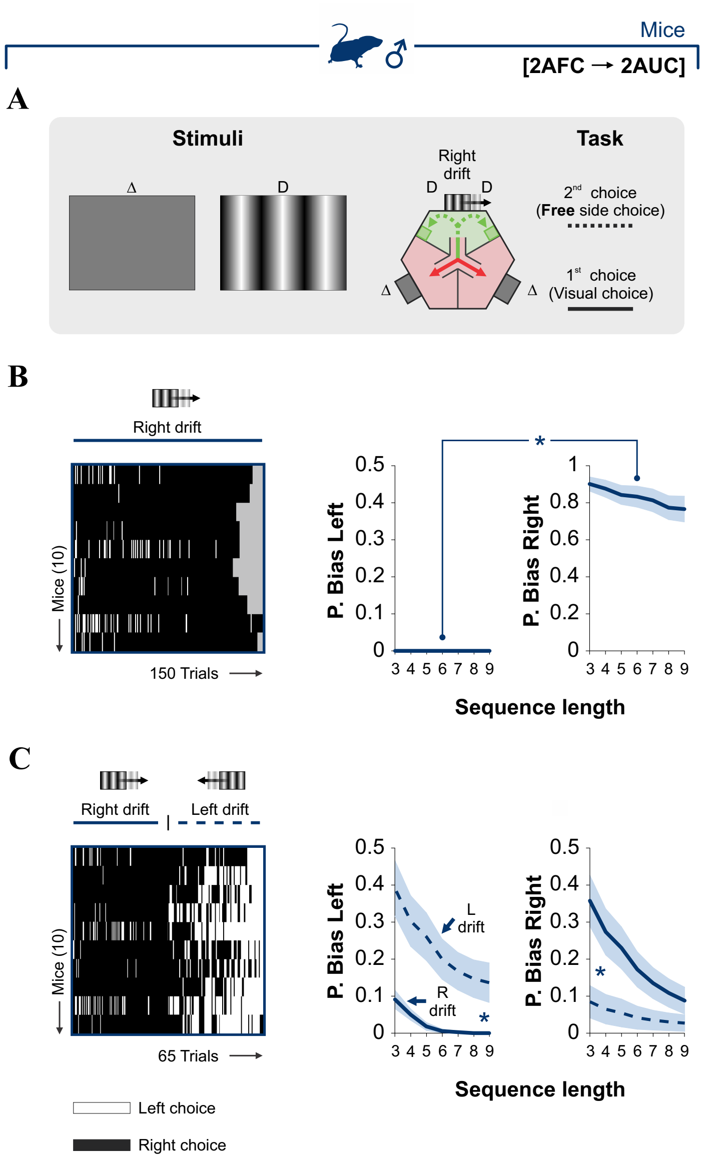

Supplementary Figure 3

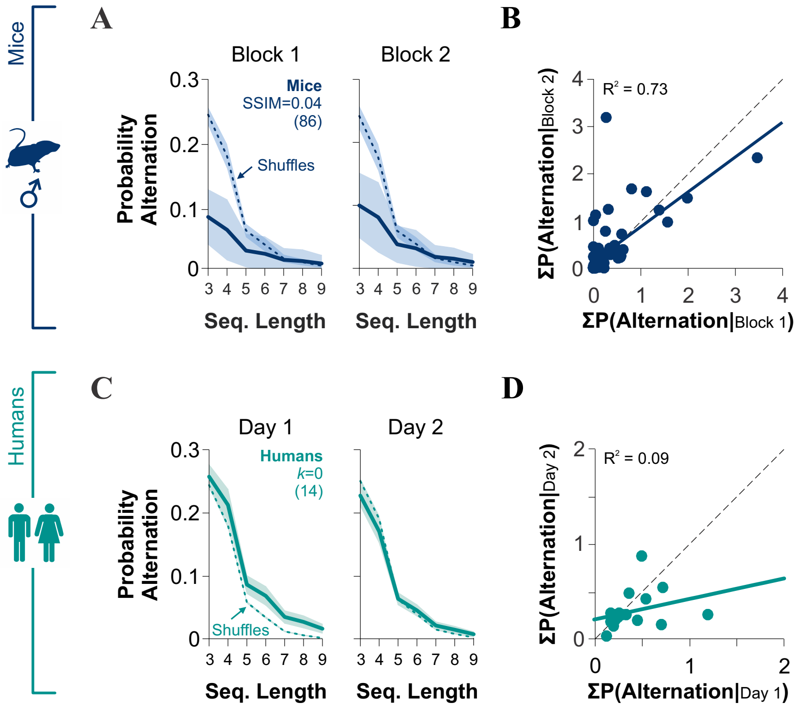

Supplementary Figure 4

Mice

A

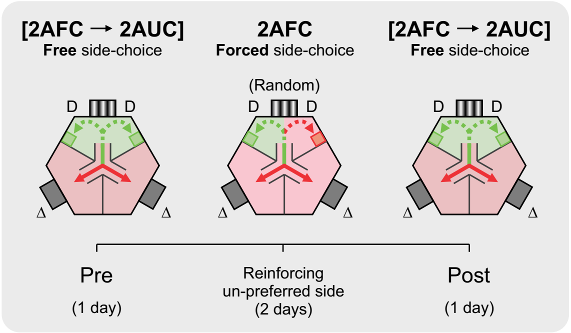

B

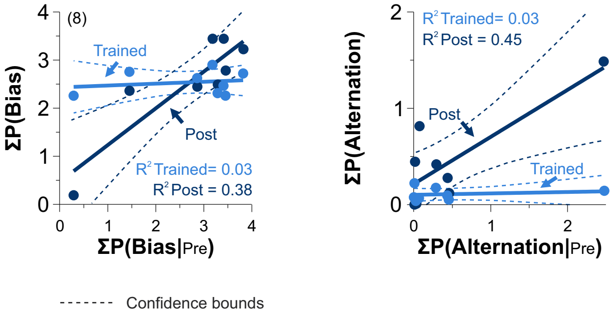

Supplementary Figure 5

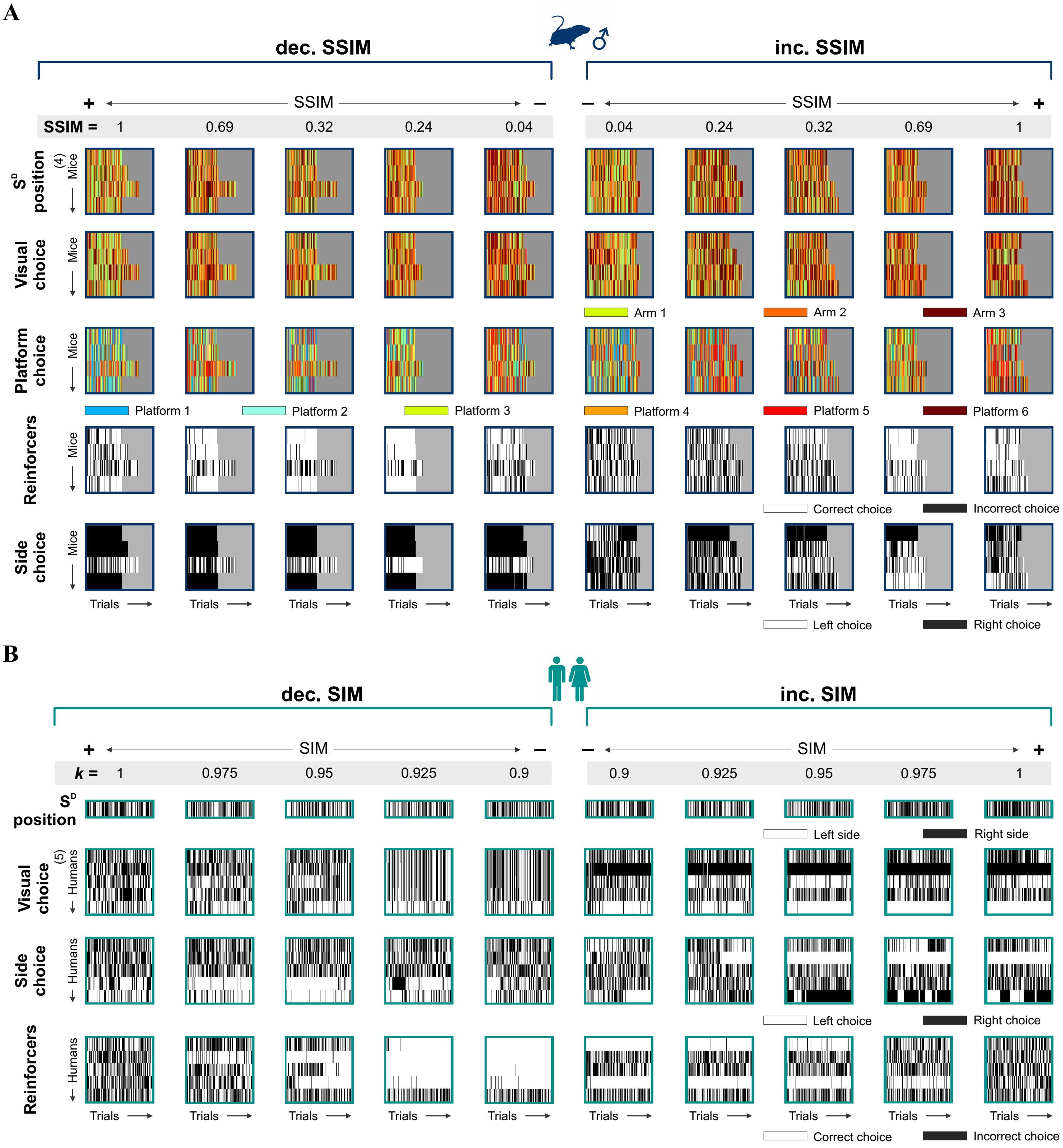

Supplementary Figure 6

2AFC
Mice

	dec. contrast (*n* =5)		inc. contrast (*n* =5)	
Contrast (%)	% Correct	Escape latency (s)	% Correct	Escape latency (s)
0.00	36.09 ± 3.28	33.18 ± 5.46	52.84 ± 0.42	36.53 ± 5.68
12.50	68.39 ± 5.24	20.16 ± 3.17	82.06 ± 4.54	11.72 ± 3.83
25.00	90.17 ± 1.79	12.71 ± 2.16	93.74 ± 2.71	10.27 ± 0.69
37.50	97.24 ± 0.59	6.85 ± 0.55	97.62 ± 1.14	8.29 ± 0.93
100.00	98.90 ± 0.40	5.80 ± 0.47	98.62 ± 0.07	6.50 ± 1.43

Data from Figure 2B

2AFC
Humans

	inc. SIM (*n* =7)		dec. SIM (*n* =7)	
k	% Correct	Reaction time (s)	% Correct	Reaction time (s)
1.000	50.44 ± 0.54	0.92 ± 0.03	50.25 ± 0.68	1.67 ± 0.12
0.975	49.68 ± 0.07	0.98 ± 0.06	61.17 ± 6.48	1.26 ± 0.16
0.950	58.38 ± 6.90	0.95 ± 0.02	67.37 ± 9.39	1.07 ± 0.12
0.925	61.58 ± 8.37	1.13 ± 0.08	66.08 ± 8.11	1.02 ± 0.11
0.900	76.31 ± 9.24	1.66 ± 0.13	58.01 ± 5.92	1.02 ± 0.09

Data from Figure 2F

Supplementary Table 1

[2AFC→2AUC]
Mice

	inc. SSIM (n=4)		dec. SSIM (n=4)	
SSIM	% Correct	Escape latency (s)	% Correct	Escape latency (s)
1.00	55.00 ± 8.56	21.88 ± 4.81	52.88 ± 10.63	11.34 ± 3.10
0.69	55.91 ± 8.25	20.64 ± 6.71	75.74 ± 6.49	9.66 ± 2.24
0.32	68.11 ± 8.29	15.43 ± 4.54	74.25 ± 12.94	11.44 ± 4.90
0.24	91.70 ± 7.58	9.13 ± 2.42	78.75 ± 12.54	8.74 ± 3.39
0.04	90.27 ± 6.83	7.25 ± 1.26	70.57 ± 12.45	14.33 ± 3.01

Data from Figure 3B

[2AFC→2AUC]
Humans

	inc. SIM (n=5)		dec. SIM (n=5)	
k	% Correct	Reaction time (s)	% Correct	Reaction time (s)
1.000	50.10 ± 5.80	2.63 ± 0.61	49.73 ± 13.26	2.47 ± 0.16
0.975	56.62 ± 7.93	2.23 ± 0.51	58.88 ± 13.24	2.33 ± 0.35
0.950	71.32 ± 11.38	1.68 ± 0.42	68.46 ± 12.86	1.85 ± 0.31
0.925	88.72 ± 10.39	1.34 ± 0.38	70.18 ± 7.72	1.94 ± 0.37
0.900	89.40 ± 10.76	1.16 ± 0.36	69.50 ± 0.59	2.21 ± 0.29

Data from Figure 3F

Supplementary Table 2

Reinforcing side-biases and alternating strategies

Mice (*n* =8)

Block	Correct (%)	Escape latency (s)
1	96.81 ± 2.44	9.32 ± 0.87
2	95.83 ± 3.15	8.05 ± 0.71
3	96.77 ± 2.05	7.92 ± 0.62
4	98.02 ± 1.55	7.31 ± 0.48
5	98.23 ± 1.43	7.15 ± 0.49
6	97.99 ± 1.79	8.29 ± 0.72

Data from Figure 7B

Humans (*n* =21)

Block	Correct (%)	Reaction time (s)
1	70.40 ± 18.06	1.65 ± 0.59
2	79.20 ± 16.50	1.30 ± 0.52
3	81.35 ± 13.21	1.17 ± 0.40
4	86.67 ± 15.80	1.00 ± 0.33
5	83.25 ± 14.78	1.17 ± 0.38
6	80.71 ± 12.64	1.09 ± 0.30
7	85.48 ± 14.48	0.95 ± 0.23
8	86.03 ± 12.75	0.92 ± 0.23
9	83.89 ± 12.03	1.05 ± 0.29
10	89.21 ± 11.98	0.87 ± 0.30
11	86.59 ± 8.29	1.04 ± 0.22
12	89.84 ± 10.08	0.84 ± 0.25

Data from Figure 7F

Supplementary Table 3
